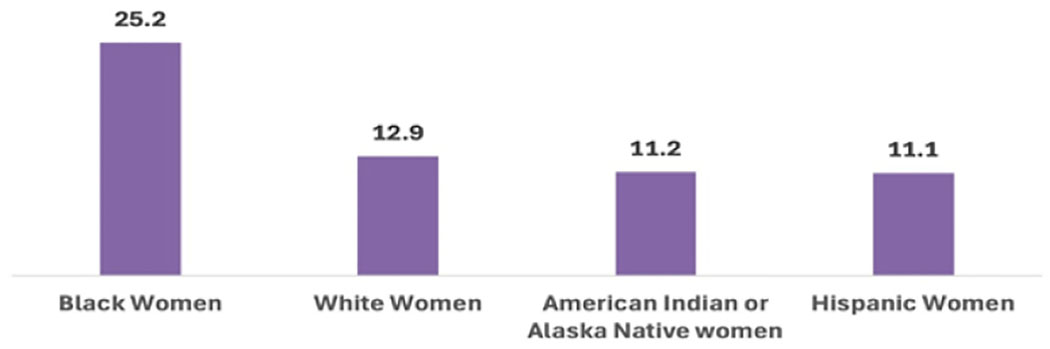# Initial Evaluation of Dihydroartemisinin (DHA), Metformin and Taro Extract in Combination with Cisplatin for Enhanced Cytotoxicity in Triple-Negative Breast Cancer (TNBC) Cell Lines

**DOI:** 10.30683/1927-7229.2026.15.06

**Published:** 2026-06-04

**Authors:** Ada Morgan Godwin, Na’Turie Ward, Christopher Krauss, Hirendranath Banerjee, Gloria Payne, Dolapo Adedeji

**Affiliations:** 1 Department of Health and Human Studies – Pharmaceutical Sciences Program, Elizabeth City State University, Elizabeth City, NC 27909, USA; 2 Department of Natural Sciences – Biology Graduate Program, Elizabeth City State University, Elizabeth City, NC 27909, USA

**Keywords:** Cisplatin, cytotoxicity, dihydroartemisinin, metformin, taro extract, triple-negative breast cancer, combination

## Abstract

This study evaluated the antiproliferative effects of cisplatin, dihydroartemisinin, metformin and taro extract on MDA-MB-231 triple-negative breast cancer (TNBC) cell lines. Cisplatin at half maximal inhibitory concentration, IC_50_ (20 μM) induced ~45% cell death (p < 0.001 vs control), while metformin (MET), dihydroartemisinin (DHA), and taro extract (TE) alone showed minimal cell death. Combination treatments (Cis + MET, Cis + DHA, and Cis + TE) significantly increased cytotoxicity to ~60% cell death on average (p < 0.01 vs cisplatin alone), with Cis + TE producing the greatest effect (~90%). The triple metabolic combination without cisplatin caused less than10% cell death (p < 0.001 vs cisplatin-containing groups), probably supporting a cisplatin-dependent cell death.

## INTRODUCTION

**Figure 1:**
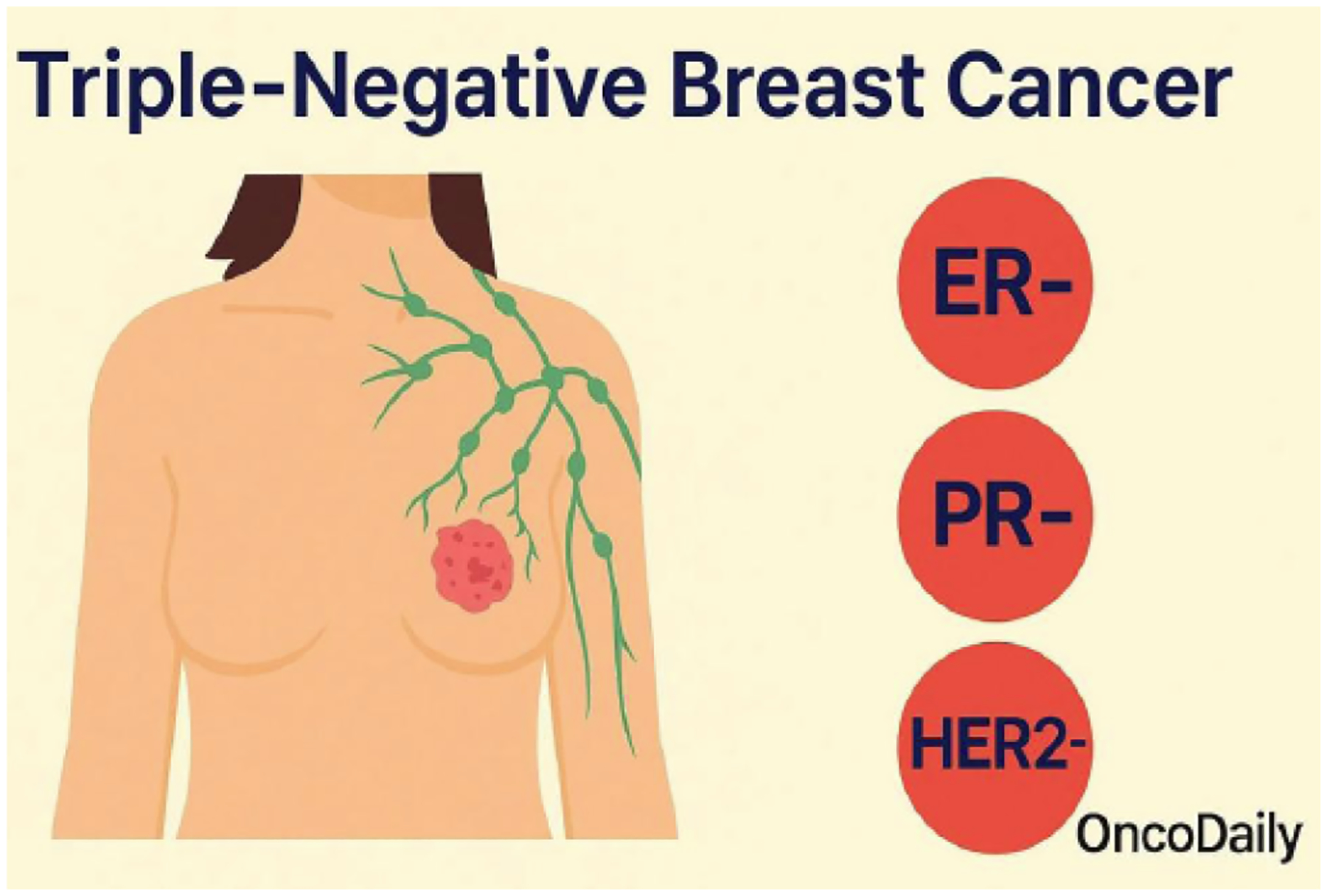
TNBC - no estrogen, progesterone and HER2.

Triple-negative breast cancer (TNBC) is an aggressive form of breast cancer that grows and spreads rapidly, disproportionately affecting Black women under 40 (Table 1) [1,2]. This accounts for 10–15% of breast cancer cases. TNBC (Figure 1) has fewer treatment options compared to other invasive breast cancer since the cancer cells lack the estrogen or progesterone receptors or enough HER2 protein to make the hormone therapy or HER2-targeted drugs work [3–5]. Metformin, a first-line for the treatment of Type 2 diabetes mellitus has shown promising anticancer properties in various malignancies, including TNBC. Studies have shown that this anti-diabetic drug exerts its antitumor effects by reduction in TNBC cell viability and induces apoptosis. Evidence also suggests that metformin targets breast cancer stem cells by reducing the resistance to chemotherapy and reoccurrence of tumors. [6–8]. Dihydroartemisinin (DHA) is a semisynthetic derivative of artemisinin developed for the treatment of malaria. Evidence suggests that DHA possess significant anti-cancer property by increasing oxidative damage and apoptosis in different cancer cell lines. DHA induces reactive oxygen species mediating oxidative stress and apoptosis in cancer [9,10]. Taro extract (TE), a natural product, has shown potential against TNBC, Taro (*Colocasia esculenta*) contains several bioactive compounds including lectins, polysaccharides and antioxidants that have shown anticancer and immunomodulatory activities. TE studies have shown to significantly inhibit tumor metastasis and reduced cancer stem cell activity in murine models [11–13]. Cisplatin, a platinum-containing drug used in the treatment of solid tumors has been shown to be efficient in the treatment of TNBC though the therapy is associated with several side effects like nephrotoxicity, neurotoxicity, ototoxicity and acquired resistance [14–16]. This initial study evaluated the anti-proliferative effects of metformin (MET), dihydroartemisinin (DHA), and taro extract (TE), alone and in combination with cisplatin, on TNBC cell lines to determine whether cisplatin doses, and related side effects—can be reduced. We hypothesized that targeting metformin, DHA, and the bioactive compounds in taro extract would enhance the cytotoxic effects of cisplatin, thereby increasing cell death in TNBC cells.

## MATERIALS AND METHODS

### Cell Culture

TNBC cell line, MDA-MB-231, purchased from American Type Culture Collection (ATCC). This cell line was selected because it has mutations (KRAS p. Gly13Asp, BRAF, NF1, and TP53) that increase drug resistance and tumorgenicity, making it ideal for the study of TNBC [17]. TNBC cells were cultured and maintained in EMEM (ATCC) media supplemented with 10% Fetal Bovine Serum (ATCC), 0.01mg/mL human recombinant insulin (Thermo Fisher Scientific, Inc.), 10ug/mL blasticidin S insulin (Gibco, Inc.) and penicillin-streptomycin (1%) (Thermo Fisher Scientific, Inc.) at 37°C in 5% CO_2_ humidified incubator. Cells were harvested upon 70–80% (about 3–4 days, with medium change every 48 hours) confluency and seeded into 96-well plates. If not use, cells were stored in 5% DMSO freezing media.

### Reagents

Cisplatin, DHA and MET were purchased from Thermo Fisher Scientific, Inc. Cisplatin and DHA were dissolved in 100% dimethyl sulfoxide (DMSO) to create a100 mM stock solution; these were stored at −20°C. Dilutions were made to 10 μM for assay. Metformin was dissolved in fresh media to create a 1 mM and then diluted to 10 μM. As reported previously, Taro extract was commercially purchased and cleaned with deionized water, peeled and cut into small pieces. The taro was then combined with PBS in a weight; volume ratio of 1:3 and blended at low speed, followed by high speed to liquefy. The blended taro was then centrifuged at 1200 r.p.m. for 15 minutes at 4°C [18].

### Cell Viability Assay

The CellTiter 96^®^ AQ_ueous_ One Solution Cell proliferation assay (Promega) was used to assess the cytotoxic properties of cisplatin, DHA, metformin and TE after 72-hour exposure. Cells were seeded at 5,000 cells per well and were given 24 hours to attach to the wells. After 24-hour incubation period, the EMEM media was aspirated and replaced with 100 ul of drug concentration range of 1 nM to 100 μM in fresh medium for cisplatin. 10 μM dihydroartemisinin and DHA, and taro extract (25 μ g/mL) were used in the experiment. These concentrations were selected based on previously reported *in vitro* dose–response studies. Metformin and DHA exhibit anticancer activity predominantly in the low micromolar range (1–25 μM), with 10 μM commonly used as a biologically active yet sub-cytotoxic concentration suitable for mechanistic and combination studies [19]. Taro (*Colocasia esculenta*) extract demonstrates dose-dependent cytotoxic effects in cancer cell lines, with reported IC_50_ values ranging approximately from 20–50 μg/mL; the25 μg/mL was selected as a sub-IC_50_ concentration to evaluate potential synergistic interactions without inducing excessive single-agent toxicity [18].

The blank row contained wells of cells, and the control contained wells of cells, and the control row contained cells and 0.001% DMSO. Three replicates of each drug concentration [cisplatin, metformin, DHA and TE alone and in combination (Cis + Met, Cis + DHA, Cis + TE)], in addition to the blank and control were run in parallel and incubated for 72 hours. Cell viability was determined quantitatively by adding tetrazolium compound [3-(4,5-dimethylthiazol-2-yl)-5-(3-carboxymetho-xyphenyl)-2-(4-sulfophenyl-)-2H-tetrazolium, inner salt; MTS] and an electron coupling reagent (phenazine ethosulfate; PES). Assays were performed by adding 10ul of the MTS reagent mixture to culture wells and then incubated for 3–4 hours in a humidified 5% CO_2_ atmosphere. The absorbance was recorded at 490 nm using a 96-well plate reader (GloMax Discover, Promega). Cell viability was determined by comparing the optical density (OD) of the treated versus the control (Corrected OD of the treated/Corrected OD of control x 100).

### Cell Death Morphology

1 × 10^5^ cells were seeded into a six-well plate. The cells were allowed to attach to the wells for 18–24 hours. Drugs were added alone and in combination - cisplatin, metformin, DHA and TE alone and in combination (Cis + Met, Cis + DHA, Cis + TE). Pseudo Confocal microscope (Keyence Corporation of America) was used to evaluate the morphological changes in treated and untreated cells.

### Statistical Analysis

The results are presented as the means ± standard deviation from three independents experiments. Experiments were performed in triplicates and data analyzed using one-way ANOVA followed by Tukey’s post hoc test. P < 0.05 was considered statistically significantly difference.

## RESULTS

**Figure 2:**
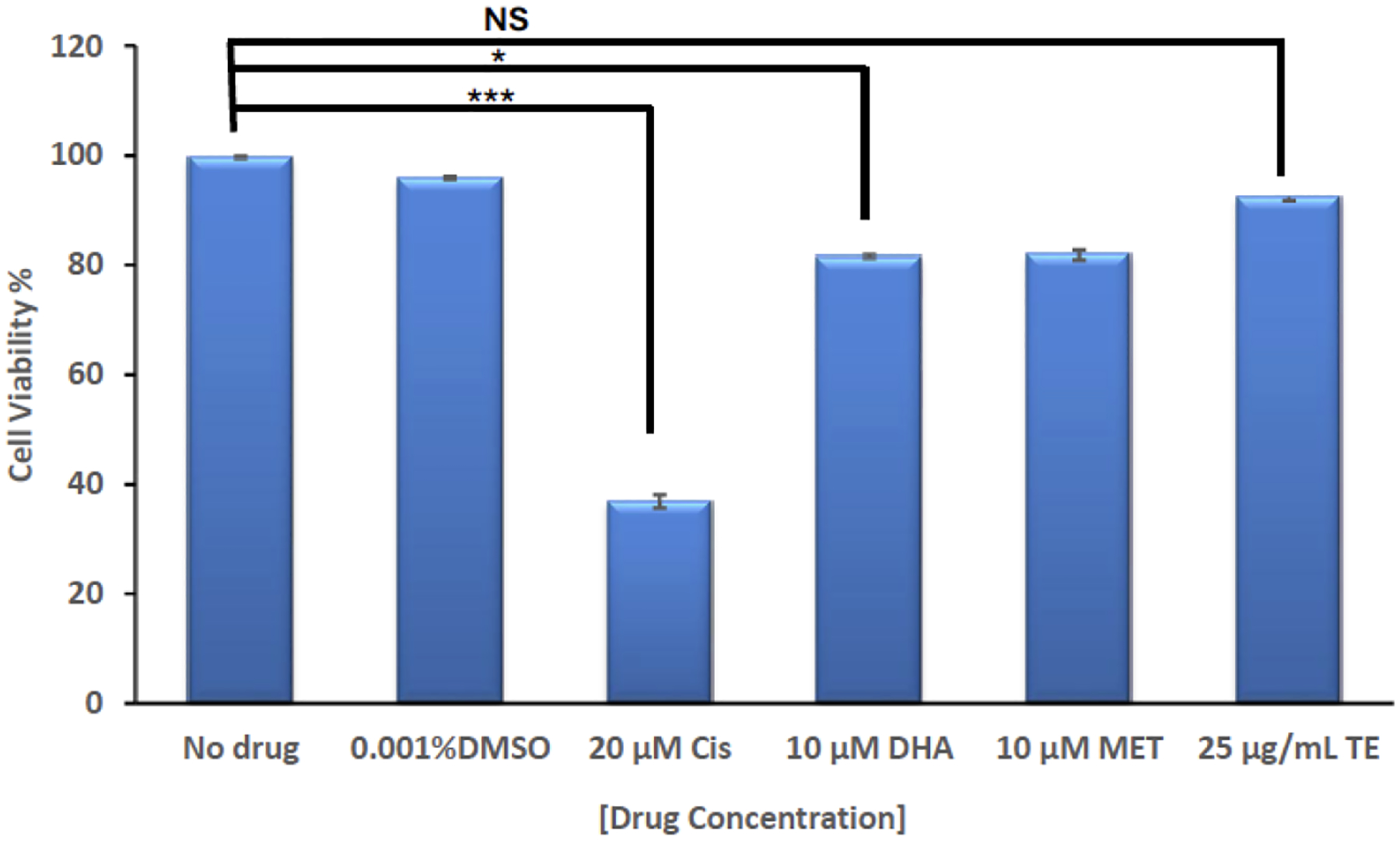
TNBC cell lines treated singly with Cis, DHA, Met and TE for 72 hrs. and viability measured by MTS assay. Data shown are mean ± SEM from three independent experiments performed in triplicate. NS= not significant; *P< 0.05; ***P<0.001.

**Figure 3:**
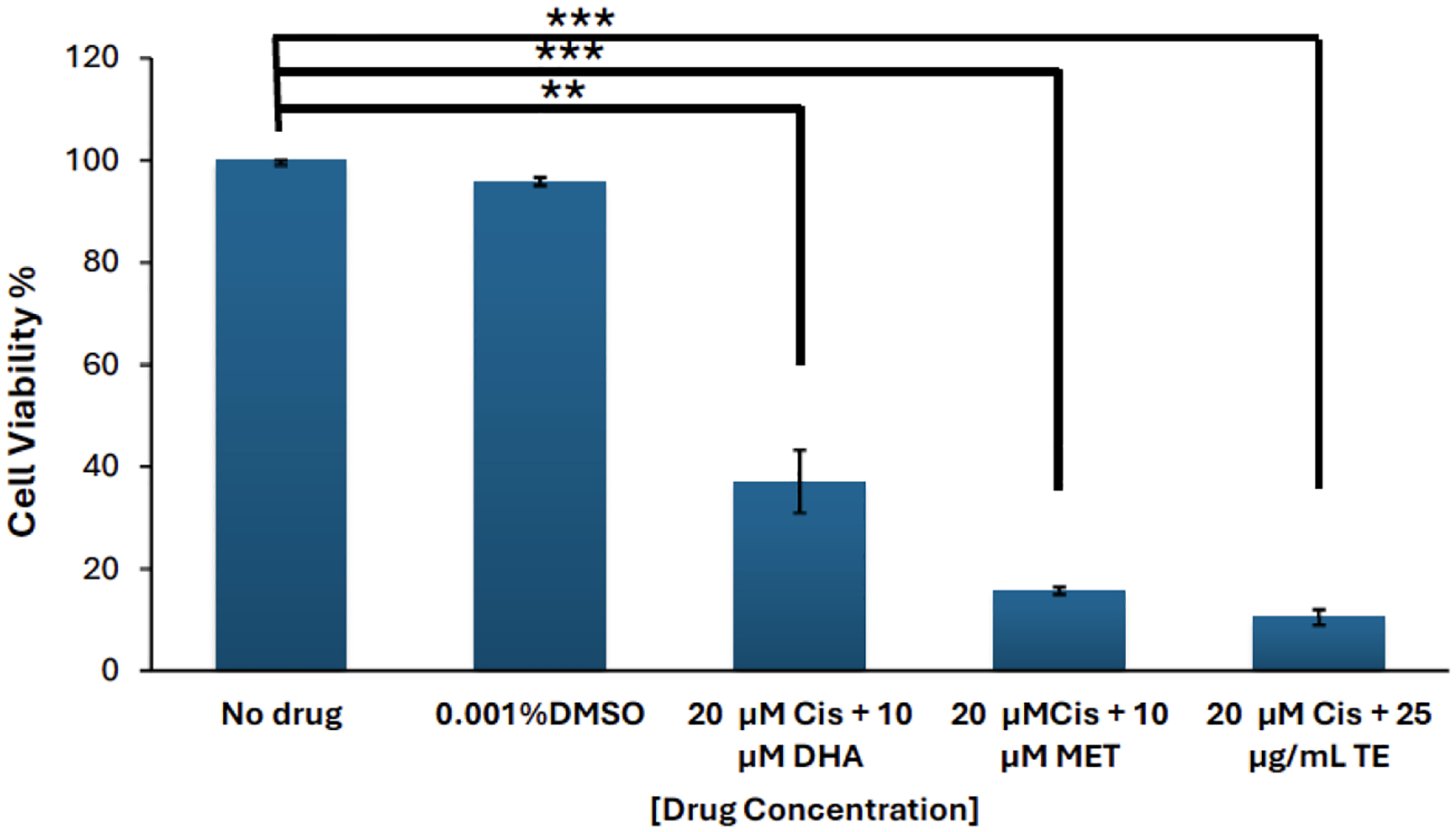
TNBC cell lines treated with Cis in combination with DHA, Met and TE for 72 hrs. and viability measured by MTS assay. Data shown are mean ± SEM from three independent experiments performed in triplicate. **P < 0.01; ***P<0.001.

**Figure 4:**
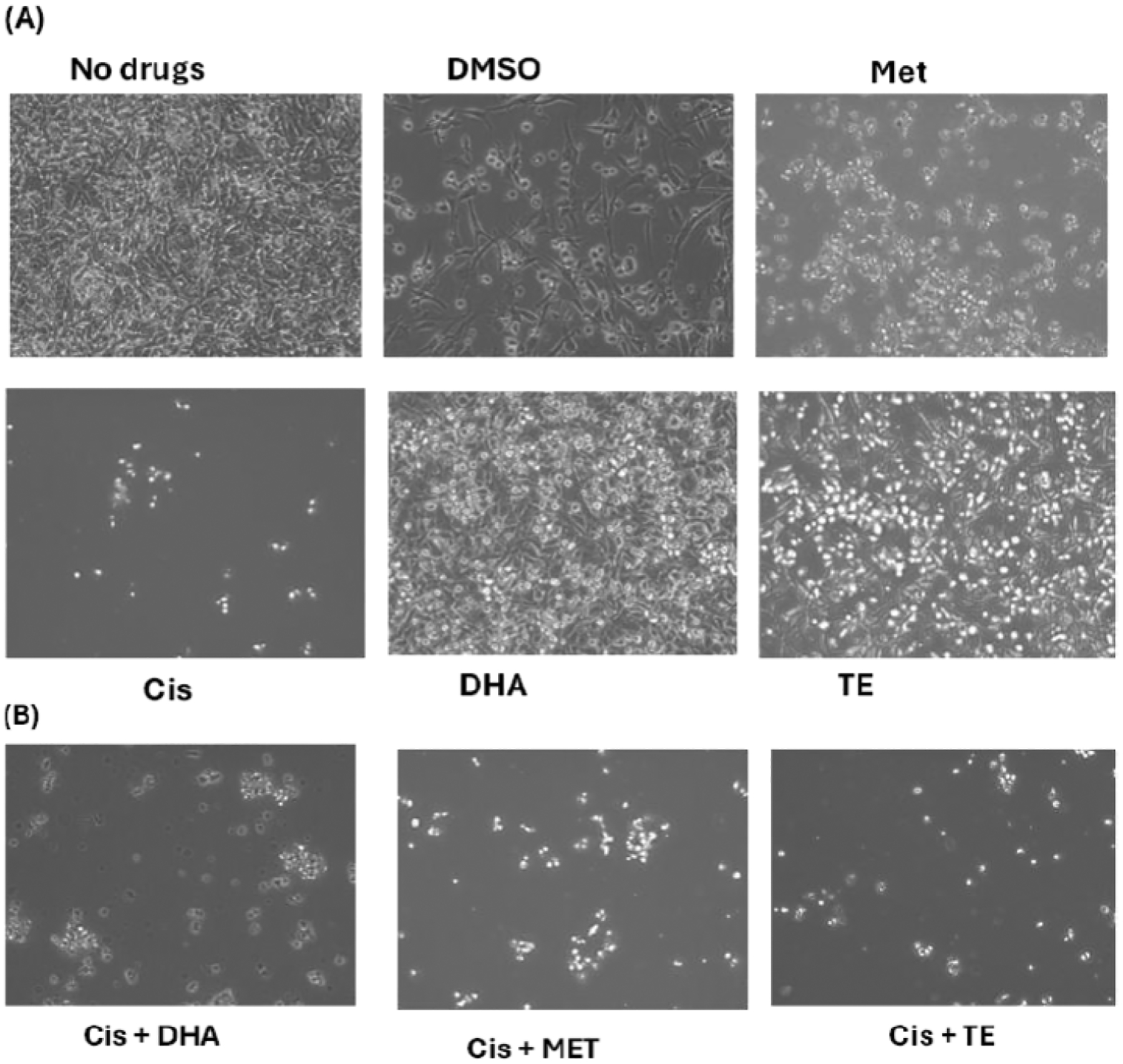
Panel **A** (single drugs) and Panel **B** (combination drugs): Images of cells captured by Pseudo Confocal microscope after 72 hrs. exposure.

The effect of cisplatin, DHA, metformin and TE on viability of TNBC cells was determined using MTS assay. First, TNBC cell lines were treated with concentrations of cisplatin ranging from 1 nM to 100 μM for 72 hrs. Based on an IC_50_ of ~40 μM (72 h), a sub-IC_50_ concentration of cisplatin (20 μM) was selected to assess combinatorial enhancement. This is to determine whether whether cisplatin doses and related side effects can be reduced. Cisplatin alone induced ~45% cell death at 72 h (p < 0.001 vs control). MET, DHA, and TE individually produced approximately 5–20% cell death (95 – 80% cell viability), with 25 μg/mL TE showing the lowest effect (~5%) (Figure 2). TE was not significantly different from control, whereas MET and DHA showed modest but significant effects (p < 0.05). Dual combinations (Figure 3) significantly decreased cell viability with Cis +TE showing the greatest cell death of ~90% while Cis + DHA (~67%) Cis + MET (~85%). P < 0.01 vs cisplatin alone. The triple metabolic combination without cisplatin resulted in ~10% cell death (p < 0.001 vs cisplatin-containing agents, data not shown), indicating that the enhanced effect is cisplatin-dependent and consistent with pharmacologic synergy rather than additive toxicity. Pseudo Confocal microscopy (Figure 4) further confirmed increased cell death morphology in control, individual- and combination-treated cells.

## DISCUSSION

Chemotherapeutic agents are widely used to inhibit cancer cell growth; however, their clinical use is often limited by significant side effects and the development of drug resistance. In the present study, the antitumor activities of cisplatin (Cis), dihydroartemisinin (DHA), metformin (MET), and taro extract (TE) were evaluated in triple-negative breast cancer (TNBC) cells. DHA and MET alone demonstrated growth inhibitory effects against TNBC cells, whereas TE alone showed limited activity as determined by MTS assay. Notably, Cis combined with DHA, MET, or TE enhanced the reduction in tumor cell proliferation and viability compared with single-agent treatment. Although this short report provides preliminary insight into the potential of these combinations to reduce TNBC cell growth, future studies will investigate whether the enhanced cytotoxicity is synergistic or additive and further evaluate mechanisms of apoptosis, including caspase activation, DNA damage, and reactive oxygen species (ROS) generation. In addition, the effects of these treatments on 3D tumor spheroid growth will be assessed.

## CONCLUSION

This study demonstrated that Cis in combination with DHA, MET, and TE reduced cell viability in TNBC cell lines. These findings highlight the potential of drug repositioning strategies using existing agents as promising therapeutic approaches for TNBC treatment without the need for new drug development.

## Figures and Tables

**Table 1:** Incidence rates of TNBC per 100,000 women in the U.S.